# First report of F1534C kdr mutation in deltamethrin resistant *Aedes albopictus* from northern part of West Bengal, India

**DOI:** 10.1038/s41598-022-17739-2

**Published:** 2022-08-11

**Authors:** Manas Pratim Modak, Dhiraj Saha

**Affiliations:** Insect Biochemistry and Molecular Biology Laboratory, Department of Zoology, University of North Bengal, Raja Rammohunpur, Siliguri, District-Darjeeling, West Bengal 734013 India

**Keywords:** Zoology, Health care

## Abstract

Dengue is the most rapidly spreading vector-borne disease with an estimated 100–400 million cases each year. Control of Dengue vectors largely depends upon synthetic pyrethroids. Development of insecticide resistance in *Aedes* mosquitoes however, poses severe threat to insecticide-based vector management programme. Mutations in the Voltage Gated Sodium Channel gene (*vgsc*) serve as the primary machinery behind this resistance development. In *Aedes albopictus*, at least four such kdr (knock down resistance) mutations had already been documented. Here, we describe the occurrence of F1534C kdr mutation in wild population of *Ae. albopictus* from northern part of West Bengal, India including a novel T1520I mutation. Four populations of *Ae. albopictus* from the studied region were found resistant against DDT and synthetic pyrethroids, among them only one population possessed F1534C kdr mutation. A total of 200 successful amplification followed by partial sequencing of *vgsc* gene further revealed the presence of F1534C kdr mutation in both phenotypically susceptible and resistant mosquito specimen. Studied populations were found 81% homozygote susceptible (1534F/F), 12.5% heterozygote (1534F/C) and 6% homozygote resistant (1534C/C) for F1534C kdr mutation. The findings of the current study will help to uncover the mechanisms underlying insecticide resistance and hence to reduce errors in vector control measurements.

## Introduction

The Asian tiger mosquito, *Aedes albopictus* (Skuse) (Diptera: Culicidae), is an epidemiologically important vector for the transmission of a variety of viral infections such as Dengue fever, Chikungunya, Yellow fever, Zika etc*.* and thus it emerged as a potential threat to global public health as well as global economy, particularly in tropical and subtropical countries like India^1,2^. In the recent past *Ae. albopictus* has been linked to Zika virus infections in the large extent of India^3^. West Bengal is a Dengue endemic state of India and all four Dengue virus serotypes has been identified from this region^4^. The temperature and relative humidity in the northern part of West Bengal are ideal for the growth and reproduction of *Aedes* mosquitoes, which has led to a number of dengue epidemics in recent years^5^. Due to the emergence and spread of new Dengue serotypes and the lack of protective vaccines, vector control is the sole way for global management of mosquito borne diseases^6^. Chemical insecticides particularly synthetic pyrethroids are the prime armament in vector population reduction as it has less toxicity to mammals and less persistent in environment^7^. In India, DDT, malathion, deltamethrin, lambda-cyhalothrin, cyfluthrin, alpha-cypermethrin, bifenthrin, and cyphenothrin are used under the National Center for Vector Borne Diseases Control (NCVBDC) for control of malaria and other vector borne diseases^8^.

Mosquitoes have acquired insecticide resistance as a result of the indiscriminate application of insecticides directly targeted at them, as well as indirect exposure to insecticides sprayed on agricultural fields^9^. As the uses of chemical insecticides increased sharply, development of resistance against these insecticides occurs simultaneously. One of the major ways of gaining such resistance is the target site insensitivity in which the targeted site for insecticide is altered through point mutations. Largely used DDT and synthetic pyrethroids targets Voltage Gated Sodium Channel (*vgsc*) gene in mosquito vectors, altering its gating properties and finally producing a *knockdown* effect^10,11^. It is a transmembrane protein, present in neuronal axons, containing four homologous domains (I–IV), each with six transmembrane segments (S1–S6) with a circular radial arrangement in which a central ion pore is formed^12^. Point mutation in the transcript of these domains results in insecticidal resistance which generally termed kdr (knock down resistance) mutation.

The first kdr mutation in *Ae. albopictus* population was reported in 2011 from Singapore, where codon for Phenylalanine (TTC) at 1534 position of IIIS6 *vgsc* gene mutated to Cystine (TGC), known as F1534C kdr mutation^13^. In the same year *Ae. albopictus* populations from Florida, USA, were reported to have the TTC to TTG mutation (F1534L) at the same gene locus^14^. A multi-country (Japan, China, Singapore, USA, France, and Italy) survey was conducted between 2011 and 2014 for the analysis of II, III, IV *vgsc* domains and revealed the presence of mutations at codons 1532 and 1534 of domain III. *Ae. albopictus* populations from Italy in particular showed the unique I1532T mutation and the F1534L mutation^15^. During the year 2017–2018, studies from China and Brazil further support the importance of the F1534C and F1534L kdr mutation in the evolution of pyrethroid resistance in the wild population of *Ae. albopictus*^2,16^. Recent studies in China further added V1016G mutation in the IIS6 region of *vgsc* gene denotes spreading of kdr mutation in the *vgsc* gene of *Ae. albopictus*^17^. In India, *Ae. albopictus* populations were found to be resistant against DDT and synthetic pyrethroids with the occurrence of some synonymous mutation at *vgsc* gene but no such kdr mutation has been discovered till date^18,19^.

Given the global rise of pyrethroid resistance and emergence of kdr mutation in the vector populations it was critical to investigate the status of resistance and the presence of probable kdr mutations in Indian *Ae. albopictus* populations. The current study focuses on the screening of major kdr mutation in the wild population of *Ae. albopictus* and their role in DDT and synthetic pyrethroid resistance development in the northern parts of West Bengal, India.

## Results

### Demography of the study area

**Figure 1:**
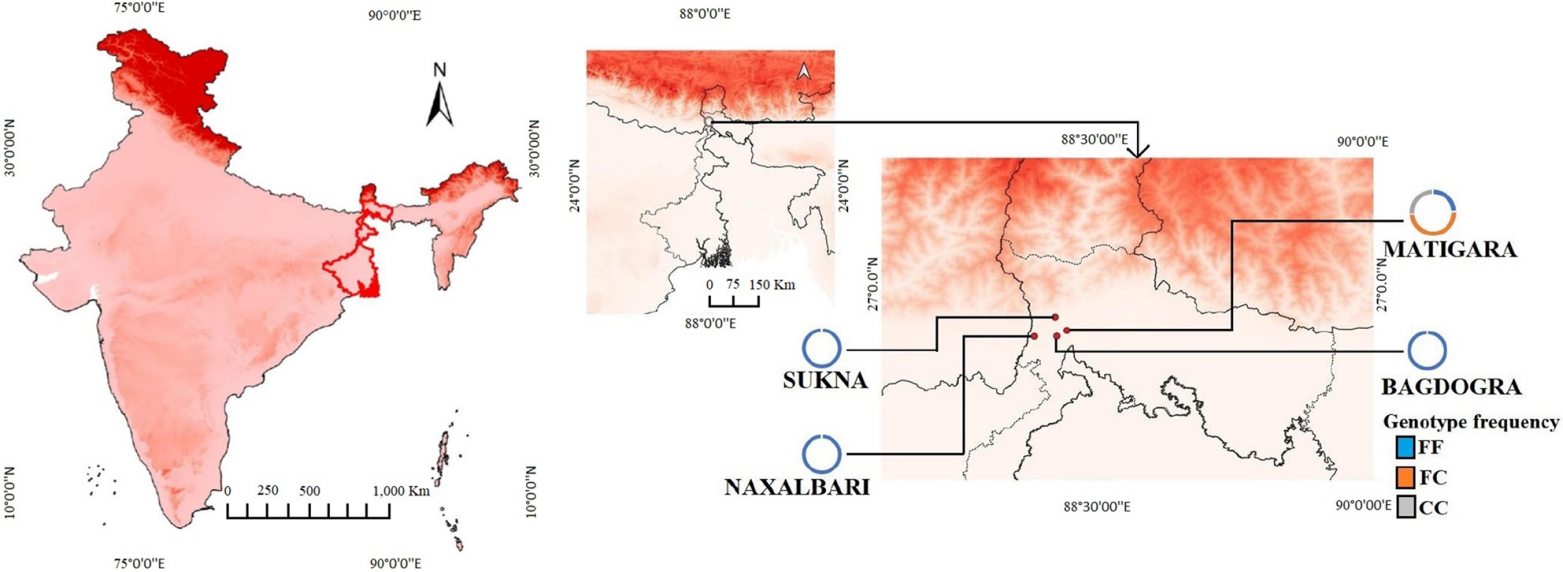
An altitude gradient map depicting the sampling site for *Ae. albopictus* from the northern part of West Bengal, India. The pie charts represent the percentage of kdr genotypes in *Ae. albopictus* population from the four different sampling site. The data were plotted on a shape file map (DIVA-GIS, https://www.diva-gis.org/gdata) using QGIS 3.16 (https://www.qgis.org/ja/site/forusers/download.html).

Darjeeling is the northernmost district of West Bengal located in foothills of Himalayas and sharing international boundaries with Nepal and Bangladesh (Fig. 1). This research was carried throughout Darjeeling district's several blocks, including rural, urban, and semi-urban areas. The average larval density at different sampling sites indicates that there are plenty of mosquito breeding habitats. Details of the mosquito collection, climatic conditions of sampling sites, co-existence of other species and nature of habitats are summarized in Supplementary Table S1.

### Insecticide resistance profile of *Ae. albopictus*

The study of adult bioassay revealed that multiple resistances were developed in the wild population of *Ae. albopictus* against DDT and synthetic pyrethroids. Mortality percentage of the studied mosquito populations against DDT, permethrin, lambda-cyhalothrin and deltamethrin was shown in Table 1.Mortality percent ranged from 75.77 to 83.30 for DDT, 86.43–93.43 for permethrin, 78.7–88.28 for lambda-cyhalothrin and 77.09–83.97 for deltamethrin. Highest level of resistance was observed against DDT in NAX^al^, BAG^al^, and MAT^al^ populations whereas SUK^al^ shows highest resistance against lambda-cyhalothrin. Mosquitoes from all the study site are confirmed resistant against all the tested insecticide except the two populations i.e., NAX^al^ and SUK^al^ were found to be possible resistant against permethrin. Among synthetic pyrethroids, lowest mortality was found in deltamethrin followed by lambda-cyhalothrin and permethrin except in SUK^al^ population where lambda-cyhalothrin showed least mortality.

**Table 1:** Insecticide resistance profile of *Ae. albopictus* (n ≥ 100) from northern part of West Bengal, India against DDT and synthetic pyrethroids.

Mosquito population	DDT M% ± S.E	Permethrin M% ± S.E	Lambda-cyhalothrin M % ± S.E	Deltamethrin M% ± S.E
NAX^al^	76.45 ± 0.43	93.15 ± 0.87	88.28 ± 0.40	77.56 ± 0.57
SUK^al^	83.30 ± 0.35	93.43 ± 1.15	78.7 ± 0.42	83.97 ± 0.41
BAG^al^	77.36 ± 0.40	88.99 ± 1.01	81.23 ± 0.41	77.09 ± 0.70
MAT^al^	75.77 ± 0.87	86.43 ± 0.49	83.76 ± 0.82	79.97 ± 1.17

M%, mortality percentage; S.E., standard error; n, total number of adult mosquitos; al, *Aedes albopictus* population.

### Knockdown rates

The knock down times (KDT_10_, KDT_50_, KDT_95_) against the tested insecticides was shown in Supplementary Table S2. NAX^al^ population showed highest KDT values against DDT. Against permethrin highest KDT_95_ value was recorded from MAT^al^ population whereas highest KDT_10_ and KDT_50_ found in NAX^al^ population. MAT^al^ population also showed highest KDT_95_ and KDT_50_ values against lambda-cyhalothrin. NAX^al^ and SUK^al^ populations had a higher KDT values against deltamethrin. Such high KDT values indicated that the various insecticides took a long time to knock down 10%, 50%, and 95% of the *Ae. albopictus* population, depicting the emergence of resistance.

### F1534C kdr genotypingof *Ae. albopictus*

**Figure 2:**
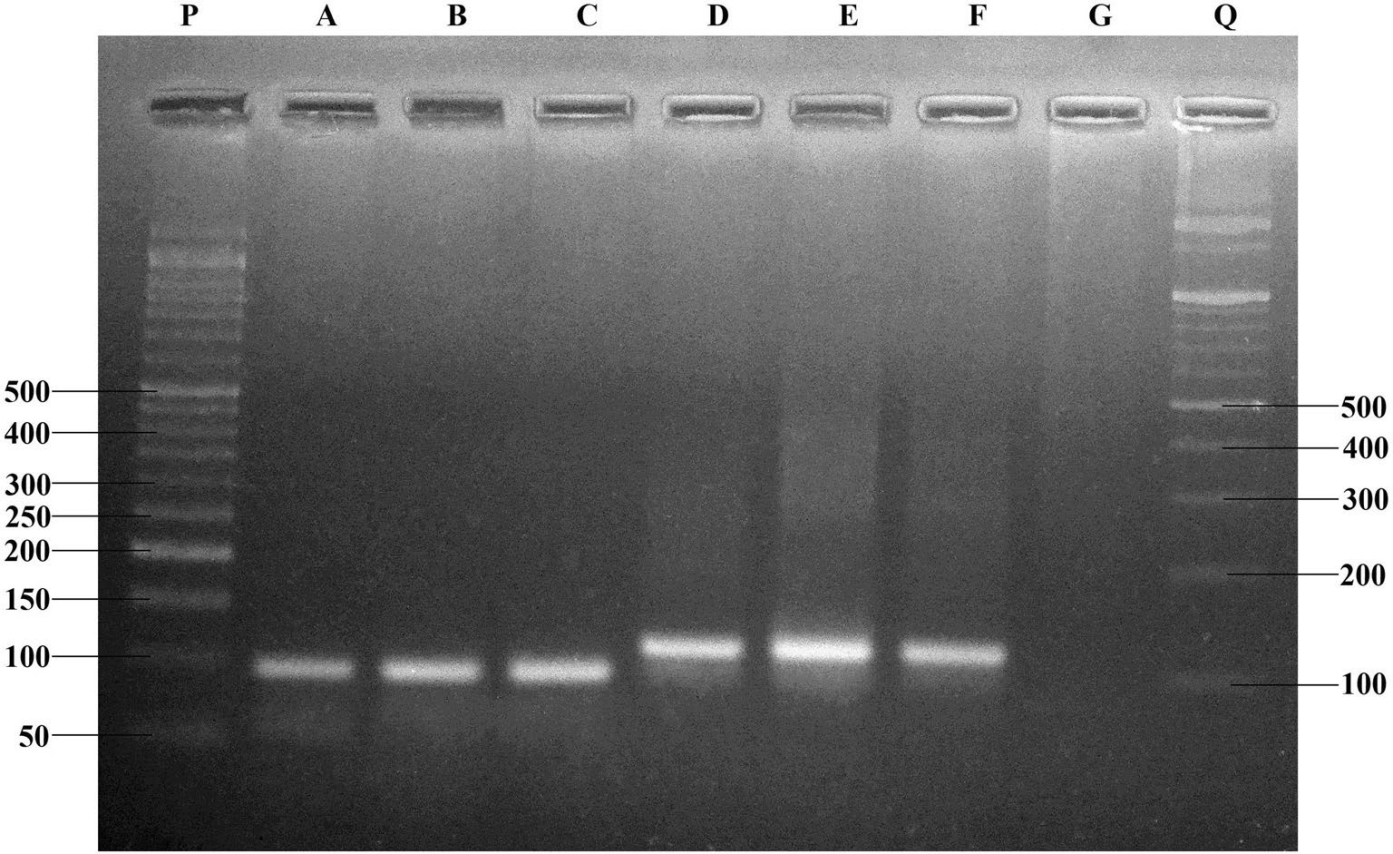
Gel electrophoresis image showing characteristic 93 and 113 bp bands obtained through allelic-specific PCR (AS-PCR) of F1534C kdr mutation in *vgsc* gene of *Ae. albopictus* from northern part of West Bengal, India. Lane P: 50–1500 bp DNA ladder, Lane Q: 100–1500 bp DNA ladder, Lane A, B: FF genotype, Lane C, D: FC genotypes, Lane E, F: CC genotype and Lane G: negative control.

**Figure 3:**
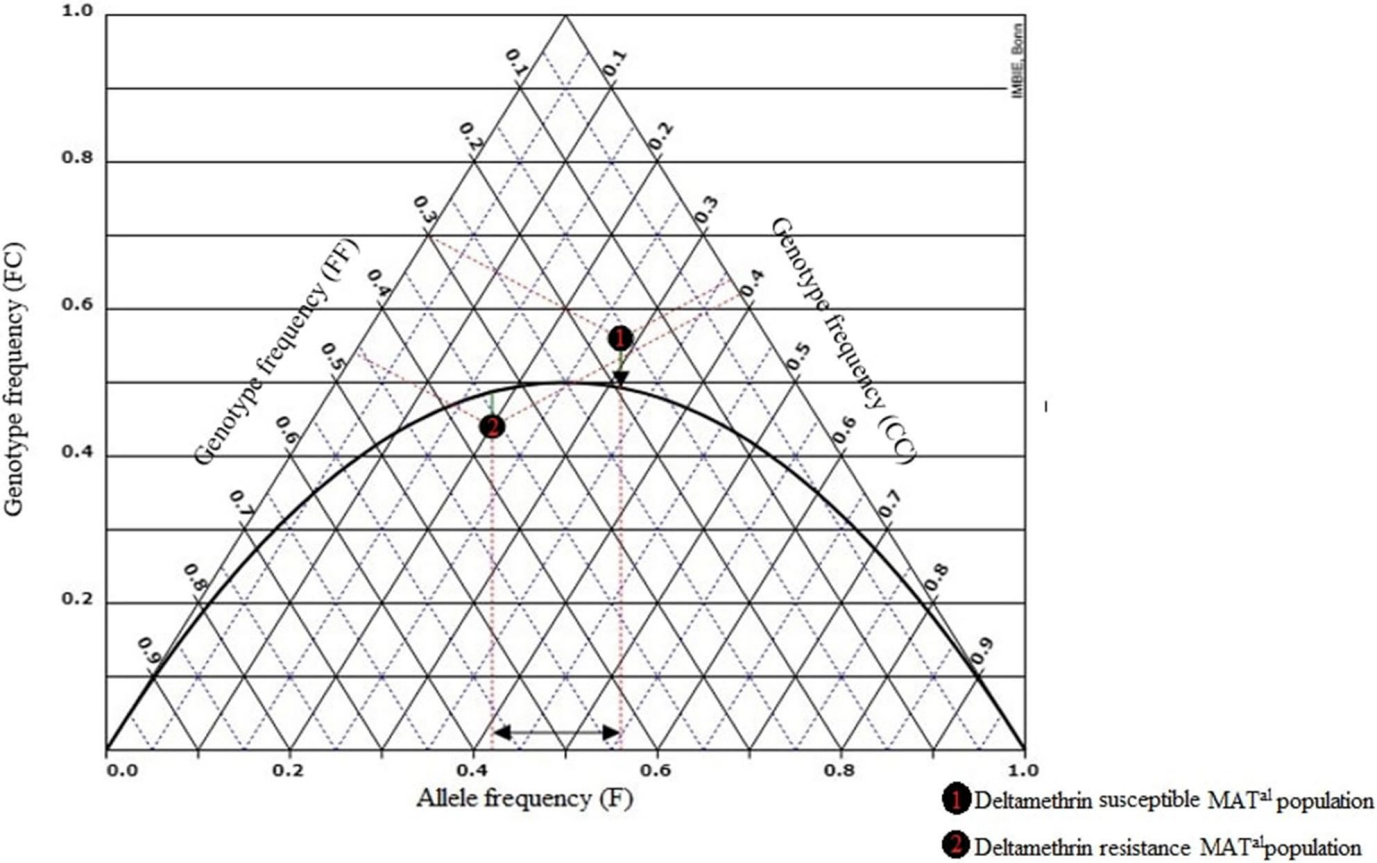
Hardy Weinberg equilibrium (HWE) parabola for deltamethrin resistant (1) and deltamethrin susceptible (2) populations of *Ae. albopictus* investigated for kdr genotypes, as shown in a De Finetti diagram. The length of the vertical line represented the frequency of genotype FC, the length of the left perpendicular line represented the frequency of genotype FF, the length of the right perpendicular line represented the frequency of genotype CC, the x-axis represented the frequency of allele ‘F’, and the Hardy–Weinberg parabola represented the point where the alleles are in Hardy–Weinberg equilibrium.

A total of 200 specimens of *Ae. albopictus* from four sampling sites was successfully amplified and all three genotypes were identified (Fig. 2). Among them, 162 (81%) were susceptible (1534 F/F), 25 (12.5%) were heterozygote (1534 F/C), and 13 (6.5%) were homozygote resistant (1534 C/C) for F1534C kdr mutation. Only one population i.e., MAT^al^ was found to carry mutated C allele (Fig. 1). The frequency of C allele in deltamethrin resistance and deltamethrin susceptible populations was found to be 0.58 and 0.44 respectively (Supplementary Table S3). Kdr genotyping demonstrate that three out of the four population i.e., BAG^al^, NAX^al^ and SUK^al^ were exclusively homozygous for F allele. In the MAT^al^ population, all three genotypes (1534F/F, 1534F/C and 1534C/C) were found for the 1534 kdr locus from both phenotypically resistant and susceptible mosquitoes. Of the three different genotypes, 50% were heterozygous (1534F/C), 26% were homozygous resistant (1534C/C) and 24% were homozygous susceptible (1534F/F). The genotype frequencies at kdr locus for deltamethrin resistance and deltamethrin susceptible population followed the Hardy Weinberg Equilibrium (HWE) (P < 0.05) (Table 2). The de Finetti diagram (Fig. 3) of genotype frequencies of MAT^al^ population reveals the exact distribution pattern of kdr genotypes and the deviation from HWE.

**Table 2:** Distribution of knockdown resistance genotypes in relation to Hardy–Weinberg proportion for deltamethrin resistance and deltamethrin susceptible *Ae. albopictus* from northern part of West Bengal, India.

MAT^al^ mosquito population	Deltamethrin resistance	Deltamethrin susceptible
**Genotypes**		
FF		
Observed No	5	7
Expected No	4.41	7.84
Chi-square (χ^2^)	0.078	0.09
FC		
Observed No	11	14
Expected No	12.18	12.32
Chi-square (χ^2^)	0.114	0.229
CC		
Observed No	9	4
Expected No	8.81	4.84
Chi-square (χ^2^)	0.004	0.145
**Allele frequency**		
F	0.42	0.56
C	0.58	0.44
**Deviation from Hardy Weinberg equilibrium**		
Inbreeding coefficient (F)	0.09688	− 0.13636
Pearson’s Chi-square (df = 1)	χ^2^ = 0.196 p-value = 0.628102	χ^2^ = 0.464 p-value = 0.495354
Exact test	p-value = 0.686793	p-value = 0.691113

### DNA sequence analysis

**Figure 4:**
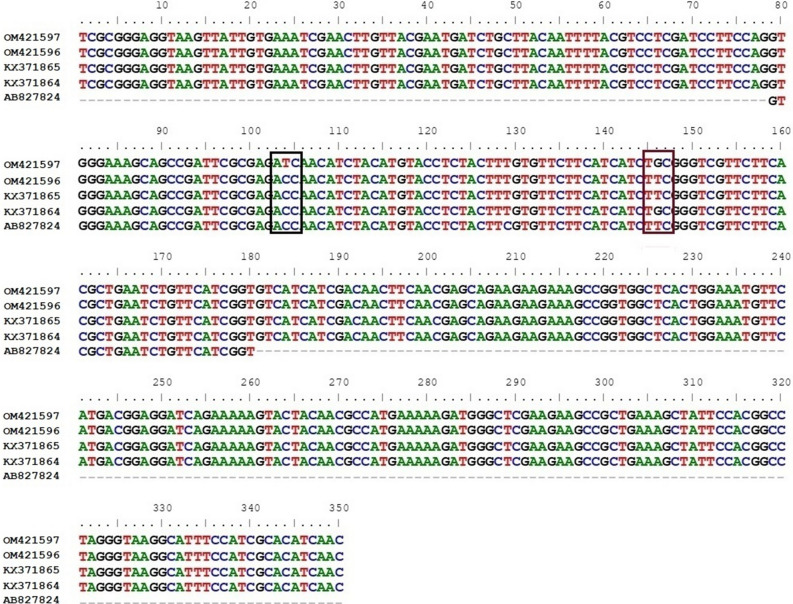
Nucleotide diversity in the IIIS6 *vgsc* gene sequence of *Ae. albopictus* from northern part of West Bengal, India. Sequence obtained from the present study (OM421596-OM421597) carried kdr mutation at 1520 and 1534 codon of IIIS6 vgsc gene, as shown black and red colour rectangular respectively. The alignment includes genomic DNA sequences of both F and C at 1534 position [1534Phe (AB827824), 1534Phe (KX371864) and 1534Cys (KX371865)] of IIIS6 *vgsc* gene, obtained from GenBank database.

An amplified 350 bp fragments (Supplementary Fig. 1) of IIIS6 *vgsc* gene were sequenced and the sequences were submitted to GenBank database (Accession No. OM421596 and OM421597). A total of six specimens from MAT^al^ population were sequenced to check efficiency of AS-PCR assay and to confirm the presence of F1534C kdr mutation in wild population of *Ae. albopictus* from India. Sequence alignment (Fig. 4) with other homologous sequences (Accession No. KX371864, KX371865 and AB827810) obtained from the study in Brazil and Japan showed the presence of both Phenylalanine (TTC) and Cystine (TGC) at 1534 codon of IIIS6 *vgsc* gene of MAT^al^ population. One out of the six samples were found to carry another mutation (T1520I) in the 1520 codon of IIIS6 *vgsc* gene i.e., Threonine (ACC) to Isoleucine (ATC) coexisting with F1534C kdr in MAT^al^ population.

## Discussion

The present study revealed that an enhanced level of insecticide resistance was prevalent among the wild populations of *Ae. albopictus* along with kdr mutation from the dengue endemic district of northern part of West Bengal, India. The occurrence of dengue cases were earlier recorded only from the plain region of northern part of West Bengal^20–22^. However, in the current study, SUK^al^ population, which has an altitude of 1400–1800 m, demonstrated strong resistance against DDT and type II pyrethroids, indicating that the higher altitude areas are at the risk of dengue outbreak. Average larval density from different sampling site gives strong support for the occurrence of dengue from this region^23^. *Armigeres sp*. was found in large number to share habitat with *Aedes* larvae especially in the higher altitude areas, which may cause Zika viral disease as it is reported from China^24^.

Incidence of several dengue outbreaks in Darjeeling district, in the recent time, gives major push to the authority for applying more insecticides (alpha-cypermethrin for adult and temephos for larvae) specially in the urban and semi urban areas of this district (personal communication). Therefore, all four *Ae. albopictus* populations in the current study, had a strong resistance against both types of synthetic pyrethroids, except the two i.e., NAX^al^ and SUK^al^ which exhibited possible resistance against permethrin. As type II pyrethroids were most frequently used for mosquito control programme in West Bengal that might reflect in the development of more resistance against type II pyrethroids as compared to type I. In addition to this, higher resistance against DDT has also been observed in all the studied populations. This could be attributed to the fact that, in India DDT is in use since 1944 till date, especially in case of vector control^25^. Such an exposure to a specific insecticide with very long residual effect for an extended period of time may have reflected in higher resistance.

Previous studies of the kdr muation in *Ae. albopictus* population from this northern part of West Bengal exhibit 11 synonymous and one non-synonymous mutations, however none of the major kdr was found^19^. Thus it was believed that an elevated level of different detoxifying enzymes i.e, Cytochromes P450 monooxygenases, Carboxylesterases and Glutathione S-transferase served the major reason behind this resistance development^5,26^. However, in the present study increase in knockdown time was observed against tested insecticides (Supplementary Table S2) which could point to the emergence of resistance in these vector populations through kdr mutation. As F1534C was the most common kdr mutation found in both *Ae. aegypti* and *Ae. albopictus*, thus allele specific PCR assay was performed for screening of this kdr mutation in wild population of *Ae. albopictus*. In comparison to *Ae. aegypti*, *Ae. albopictus* population from the study site experience low insecticide exposure because of their exophilic and exophagic nature^27^. This different insecticide pressure might be the reason behind an early gaining of F1534C kdr mutation in *Ae. aegypti* population of this same geographic area^28^. In the current study one population i.e., MAT^al^ out of the four population was found to carry the mutated C allele, with a frequency of 0.44–0.58. Partial sequencing of IIIS6 *vgsc* gene sequence from MAT^al^ population further confirmed the polymorphic site (TTC to TGC) at 1534 codon on exon no 31 of *vgsc* gene. Changes in the genetic makeup particularly in those genes dealing with resistance development might be the result of an increased insecticide selection pressure. In *Ae. albopictus* the position of 1534 at IIIS6 *vgsc* gene is very changeable, as different codons were found like like TGC for Cystine, TTG for Leucine, TCC for Serine with the wild type TTC and TTT for Phenylalanine^15,29^. A significant deviation of genotype frequencies from Hardy Weinberg Equilibrium (HWE) (Table 2) in the MAT^al^ population further confirm the presence of insecticide selection pressure in the studied vector population, as shown in the de Finetti diagram (Fig. 3).

In the current study, majority (50%) of *Ae. albopictus* from MAT^al^ population were found heterozygous for the F1534C mutation (Supplementary Table S3), indicating that the kdr mutation in these mosquito populations has only recently begun, as has been observed in other parts of the world^30^. Occurrence of both resistant and susceptible allelic genotypes from phenotypically susceptible mosquitoes makes it difficult to conclude a link between presence of kdr mutation and insecticide resistance status of the studied population. Finding from the current study contradict with the study in *Ae. aegypti* population of India, where this F1534C kdr mutation showed a positive association with DDT and deltamethrin resistance^31^. Given that kdr is a recessive trait, in deltamethrin resistant MAT^al^ population the occurrence of both heterozygous (1534F/C) and homozygous susceptible (1534F/F) *Aedes* individuals suggest that other mechanisms are also involved in resistance development. Our previous study also supports this fact that elevated levels of metabolically detoxifying enzymes are also present in *Ae. albopictus* population of northern part of West Bengal, India^5,26^. Result from the studies of kdr mutation on *Culex quinquefasciatus* population of north eastern India further support the observation of current study^32^. Sequence analysis from present study found another novel single nucleotide polymorphism at 1520 codon of *vgsc* gene (ACC to ATC) resulting T1520I kdr mutation in wild population of *Ae. albopictus* from India. Previously this mutation has not been reported in *Ae. albopictus* around the globe. However, due to scarcity of data on 1520 kdr site of IIIS6 *vgsc* gene, further analysis was not feasible. Though the role of T1520I kdr mutation is not clear but this mutation has always been identified in association with the F1534C mutation in *Ae. aegypti*^31^. Thus, the T1520I kdr mutation was hypothesized to be a compensating mutation to minimize the fitness cost of the F1534C mutation's possibly harmful effect, despite laboratory findings that *Ae. aegypti* homozygous for the F1534C mutation has no reduced fitness^33^. Future work in the target site insensitivity especially in the GABA receptor, acetylcholine esterase and also the insecticide penetration through cuticle may also give more insight in this resistance development.

Thus, findings from the present study suggest that wild population of *Ae. albopictus* from northern part of West Bengal possesses both kdr mutation and increased expression of detoxifying enzymes for resistance development against routinely used insecticides. This is due to erroneous applications of large amounts of insecticide and repeated applications of the same insecticide over a lengthy period of time. This has a strong consequence on the authority to be more cautious when using insecticides and to implement more alternative vector control tactics, such as the release of Wolbachia-infected male mosquitoes or more uses of biological insecticide such as *Bti* which are becoming increasingly crucial in the current world situation^34,35^.

## Materials and method

### Ethics statement

As the present study did not involve any human trial or higher vertebrates, the Institutional Animal Ethics Committee (IAEC) Department of Zoology, University of North Bengal (Regn. no. 840/GO/Re/S/04/CPCSEA) granted a waiver for ethics approval. The use of rat for blood feeding was also approved by the IAEC (approval no. IAEC/NBU/2019/19). All procedures were performed in accordance with relevant guidelines of the IAEC and ARRIVE (Animal Research: Reporting of In Vivo Experiments).

### Study area and sample collection

Dengue endemic Darjeeling district from northern part of West Bengal was surveyed and four different sites were selected for sampling. Larvae and pupae were collected from different breeding places and were transferred to plastic containers and brought to the laboratory. In the laboratory, mosquito larvae and pupae were reared up to F1 generation under controlled conditions (temperature 27 °C ± 2 °C; relative humidity 75% ± 10%). Standard identification key of larva and adult mosquitoes was used to identify the field population up to species level^36^. The sampling was done during June to November in 2020 and March to September in 2021. Since all the sampling was done from private land, prior permission was taken from the land owner for mosquito collection.

### Insecticides used

Insecticide impregnated papers (4% DDT, 0.05% deltamethrin, 0.05% lambda-cyhalothrin, 0.75% permethrin) used for adult bioassay were purchased from Vector control unit, Universiti Sains Malaysia.

### Insecticide susceptibility bioassay

The WHO (World Health Organization) adult bioassay protocol was followed for the detection of susceptibility status of the mosquito populations^37^. Seven replicates of 20 active 3–5 days non-blood fed female mosquitoes from each population were exposed to insecticide impregnated paper for an hour and were transferred to a retention tube containing cotton balls soaked in 10% sucrose solution and maintained at laboratory condition. For control, mosquitoes were placed in tubes containing paper impregnated with silicone oil. Mortality percentage was recorded after 24 h post-exposure and was repeated thrice for every insecticide. In order to calculate the knockdown time (KDT), knocked down mosquitoes were counted after every 10 min during 1 h insecticide exposure as per previous protocol^38^. The live and dead mosquitoes obtained from the adult bioassays were kept at − 20 °C and employed for DNA isolation.

### Extraction of genomic DNA

Genomic DNA was extracted from both the resistant and susceptible mosquitoes following the High Salt protocol with minor modifications as described previously^38^. Purity of the extracted DNA was checked by the SPECTROstarNano fast scanning UV–visible Microplate Reader (Make-BMG Labtech, Germany). DNA with an OD_260_/OD_280_ value between 1.8 and 2 was selected for kdr genotyping.

### Allele-specific PCR (AS-PCR) assay for F1534C kdr mutation

DNA stock solutions were prepared at a concentration of 25 ng/μl and used for AS-PCR genotyping. The Polymerase chain reaction (PCR) involved one reverse primer: 5′-TCT GCT CGT TGA AGT TGT CGA T-3′ used for both alleles, and two forward allele-specific primers: 1534Phe: 5′-GCG GGC TCT ACT TTG TGT TCT TCA TCA TAT T-3′ and 1534Cys ^kdr^ allele: 5′-GCG GGC AGG GCG GCG GGG GCG GGG CCT CTA CTT TGT GTT CTT CAT CAT GTG-3′ with an annealing temperature of 60 °C^16^. Each reaction was performed in a 25 μl volume consisting of 1.5 mM MgCl_2_, 1 × PCR buffer (Promega, USA), 0.25 μ Mcommon reverse primer, 0.125 μM each mutation specific primer, 200 μM dNTP mixture (Promega, USA), 0.2 units Taq polymerase (Promega, USA) and 25 ng genomic DNA. The thermal cycling condition was set with an initial DNA denaturation step for two minutes at 94 °C, followed by 35 cycles of denaturation for 30 s at 94 °C, annealing for 30 s at mentioned temperature and extension at 30 s at 72 °C. PCR amplification products were loaded onto a 4% agarose gel and run for 1 h at 100 V in TAE buffer and visualized by ethidium bromide staining under UV light. Since the primer used had GC tails of varying lengths, amplified products could be differentiated by base pair size as of 93 and 113 bp.

### Amplification and sequencing of IIIS6 *vgsc*gene of *Ae. albopictus*

PCR reaction was carried out for the partial amplification of IIIS6 *vgsc* gene with a expected length of 350 bp. Primers used for these reactions were AaEx31P (5′-TCG CGG GAG GTA AGT TAT TG-3′) and AaEx31Q (5′-GTT GAT GTG CGA TGG AAA TG-3′)^16^. Reaction was carried out with 1X Go®Taq G2 Green Master Mix (Promega, USA) of 12.5 μl, 1 μl of both forward and reverse primers, 2 μl of template DNA and 8.5 μl of nuclease free water in 25 μl reaction mixture. PCR condition was: 95 °C for 5 min, followed by 35 cycles of 95 °C for 30 s, 60 °C for 40 s and 72 °C for 1 min with a final extension step at 72 °C for 5 min. The amplified fragments of the expected size were purified using ExoSAP following manufacturer recommendations and were sequenced (Heredity Life sciences Pvt. Ltd. Patia, Bhubaneswar-751024, Odisha, India). The sequences were analyzed with BioEdit software (v 7.0.9) and aligned with different homologous regions of *vgsc* gene sequences (KX371864, KX371865, and AB827824) of *Ae. albopictus* available in GenBank databse by using ClustalW software (v 2.0)^39,40^.

### Data analysis

Mean mortality percentage against all the tested insecticides were calculated by using kyPlot 6.0. In WHO adult bioassays, control mortalities were below 10%, so no calculation of corrected mortality was needed. WHO 2016 criteria were followed to determine the resistance/susceptibility status [S = Susceptible (Mortality percentage = 98–100%); R = Confirm Resistance (Mortality percentage < 90%); PR = Possible Resistance (Mortality percentage = 90–98%)]. Knockdown times were determined by performing probit regression analysis in IBM SPSS (v21.0) at 95% confidence level. The web-based programme 'de FINETTI generator' version (v3.0.5) (2008) (https://finetti.meb.uni-bonn.de/) was used to compute genotype frequencies and their deviation from the Hardy–Weinberg equilibrium (HWE), which was shown within the de Finetti diagram. The diagram includes a triangular plot which represent the distribution of three genotypes in reference to one another. The curved line in the diagram represents the Hardy–Weinberg parabola that indicates the sites where alleles are in a state of HWE. The chi-square test is used to calculate the significance of the distance between the parabolic curve and the genotypes, which reflects the extent of divergence from the HWE.

## Conclusions

The current insecticide susceptibility status of the wild population of *Ae. albopictus* from northern part of West Bengal, India was reported in this study. Furthermore, this is the first report of the F1534C kdr mutation in the wild population of *Ae. albopictus* together with T1520I kdr mutation from India that we are aware of. The occurrence of the kdr mutation in the natural population of *Ae. albopictus* in India is a clear indication that the resistance monitoring programme should be reviewed and an alternative vector control method should be used. Findings of this study could be used as a starting point for additional research and the development of effective insecticide-based interventions against *Ae. albopictus* population of India.

## Supplementary Information

Supplementary Information.

## Data Availability

The nucleotide sequences generated and/or analyzed during the current study are available in GenBank (accession number OM421596 and OM421597). [https://www.ncbi.nlm.nih.gov/nuccore/OM421596.1/, https://www.ncbi.nlm.nih.gov/nuccore/OM421597.1/].
