# Supplementary material for: First report of F1534C kdr mutation in deltamethrin resistant Aedes albopictus from northern part of West Bengal, India
Source: Sci Rep. 2022 Aug 11;12:13653. doi: 10.1038/s41598-022-17739-2 (PMC9372028; doi:10.1038/s41598-022-17739-2)
Supplement: Supplementary file 1 — Supplementary Information. [file 41598_2022_17739_MOESM1_ESM.pdf]

First report of F1534C kdr mutation in deltamethrin resistant *Aedes albopictus* from northern part of West Bengal, India.

Manas Pratim Modak[1] and Dhiraj Saha[1]*

[1]Insect Biochemistry and Molecular Biology Laboratory, Department of Zoology, University of North Bengal. District - Darjeeling, West Bengal, India, 734013.

*dhirajsaha.nbu@gmail.com; dhirajsaha@nbu.ac.in

Supplementary Table S1. Sampling details of *Aedes albopictus* from northern part of West Bengal, India.

Sampling Site	Geographical coordinates	Population Name	Average larval density/500ml	Nature of habitat	Co-existence of other species	Area types	Average annual rainfall (mm)	Average Temperature (°C)	Altitude (m)
Bagdogra	26.6850˚ N 88.3254˚ E	BAG[al]	137.5	Discarded containers, Flower pots	unidentified larvae	Semi urban	271.5 (8-893)	24.25 (12 -34)	130
Matigara	26.7166˚ N 88.3833˚ E	MAT[al]	201.5	Discarded tyres	*Ae. aegypti*, unidentified larvae	Urban	303.29 (10.63-902.02)	24.25 (12 -34)	122
Naxalbari	26.6838˚ N 88.2007˚ E	NAX[al]	107	Bamboo stump, Plastic tank	*Armigeres sp. Toxorhynchites sp.*	Rural	358.12 (12.97-1087.23)	24.12 (11 -34)	164
Sukna	26.8821˚ N 88.2788˚ E	SUK[al]	109.5	Cemented tank, Discarded tyres,	*Armigeres sp.*	Rural	250.91 (10-843.9)	29.16 (18 -41)	1532

Supplementary Table S2. Knockdown time (KDT^{10}, KDT^{50} and KDT^{95}) of *Ae. albopictus* (n ≥ 100) against DDT and synthetic pyrethroids. KDT- Knock Down Time (minutes); S.D -Standard Deviation.

**Mosquito Population**	**DDT**			**PERMETHRIN**			**LAMBDA-CYHALOTHRIN**			**DELTAMETHRIN**		
	**KDT_{10}** ±S.D	**KDT_{50}** ± S.D	**KDT_{95}** ± S.D	**KDT_{10}** ±S.D	**KDT_{50}** ±S.D	**KDT_{95}** ±S.D	**KDT_{10}** ±S.D	**KDT_{50}** ±S.D	**KDT_{95}** ±S.D	**KDT_{10}** ±S.D	**KDT_{50}** ±S.D	**KDT_{95}** ±S.D
NAX[al]	20.85 ±2.10	134.66 ±0.54	490.53 ±3.99	7.25 ±0.85	45.97 ±2.23	187.87 ±2.16	4.90 ±0.29	34.17 ±1.25	136.81 ±0.633	5.22 ±0.61	47.78 ±1.09	260.97 ±2.42
SUK[al]	6.87 ±0.64	36.81 ±1.7	121.34 ±1.95	4.86 ±0.60	35.10 ±1.60	143.68 ±2.77	6.29 ±1.42	33.77 ±1.97	134.24 ±1.24	4.18 ±0.37	47.88 ±2.13	260.98 ±2.45
BAG[al]	8.18 ±0.63	42.65 ±2.35	152.61 ±1.87	6.03 ±1.96	29.81 ±2.76	106.29 ±4.18	5.67 ±2.24	34.58 ±4.98	164.10 ±3.98	3.99 ±1.62	29.37 ±2.86	156.35 ±3.34
MAT[al]	6.98 ±1.3	49.03 ±3.41	257.71 ±3.88	4.12 ±1.07	35.21 ±2.83	194.62 ±3.25	4.63 ±0.92	55.36 ±2.40	275.56 ±2.52	4.51 ±0.70	36.08 ±2.34	149.20 ±3.95

Supplementary Table S3. Genotype and allelic frequencies of F1534C kdr mutations in *Ae. albopictus* population from northern part of West Bengal, India.

Mosquito Population	Phenotype	Total PCR sample	Genotype Frequency			Allele frequency		Wright Index of inbreeding Coefficient (F_{rs})
			FF	FC	CC	F	C	
BAG[al]	Resistant	25	25	0	0	1	0	-
	Susceptible	25	25	0	0	1	0	-
MAT[al]	Resistant	25	5	11	9	0.42	0.58	0.9688
	Susceptible	25	7	14	4	0.56	0.44	-0.13636
NAX[al]	Resistant	25	25	0	0	1	0	-
	Susceptible	25	25	0	0	1	0	-
SUK[al]	Resistant	25	25	0	0	1	0	-
	Susceptible	25	25	0	0	1	0	-

Supplementary Figure 1. Gel electrophoresis image showing 350 bp band indicating amplified region of domain III segment 6 of *vgsc* gene in *Ae. albopictus*. Lane M represent 100-1500 bp DNA ladder, Lane A,B,C,D,E represent amplified IIIS6 of *vgsc* gene and Lane F represent negative control.

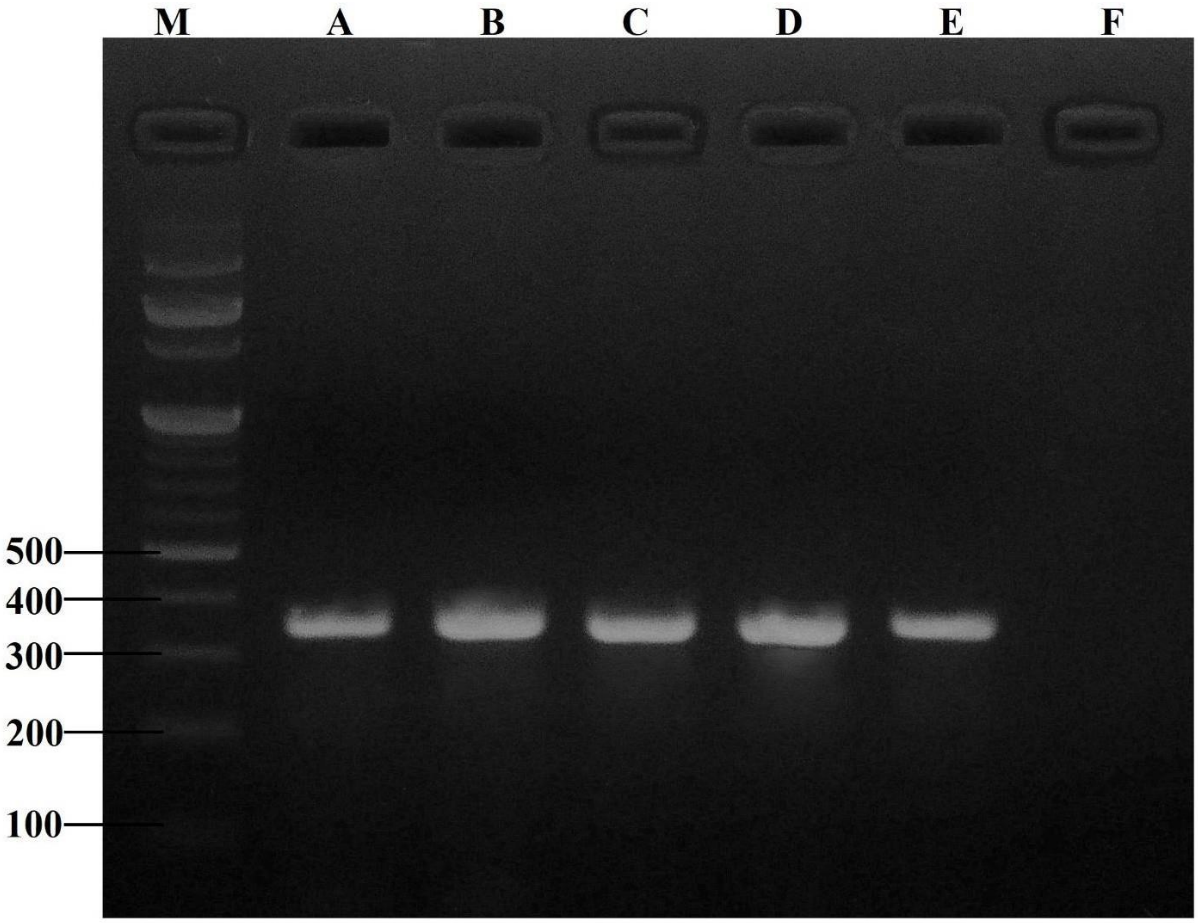

Supplementary Figure 2. Gel electrophoresis image showing characteristic 93 and 113 bp bands obtained through allelic-specific PCR (AS-PCR) of F1534C kdr mutation in *vgsc* gene in *Ae. albopictus* from northern part of West Bengal, India. Lane P: 50-1500 bp DNA ladder, Lane Q: 100-1500 bp DNA ladder, Lane A, B: FF genotype, Lane C, D: FC genotypes, Lane E,F: CC genotype and Lane G: negative control.

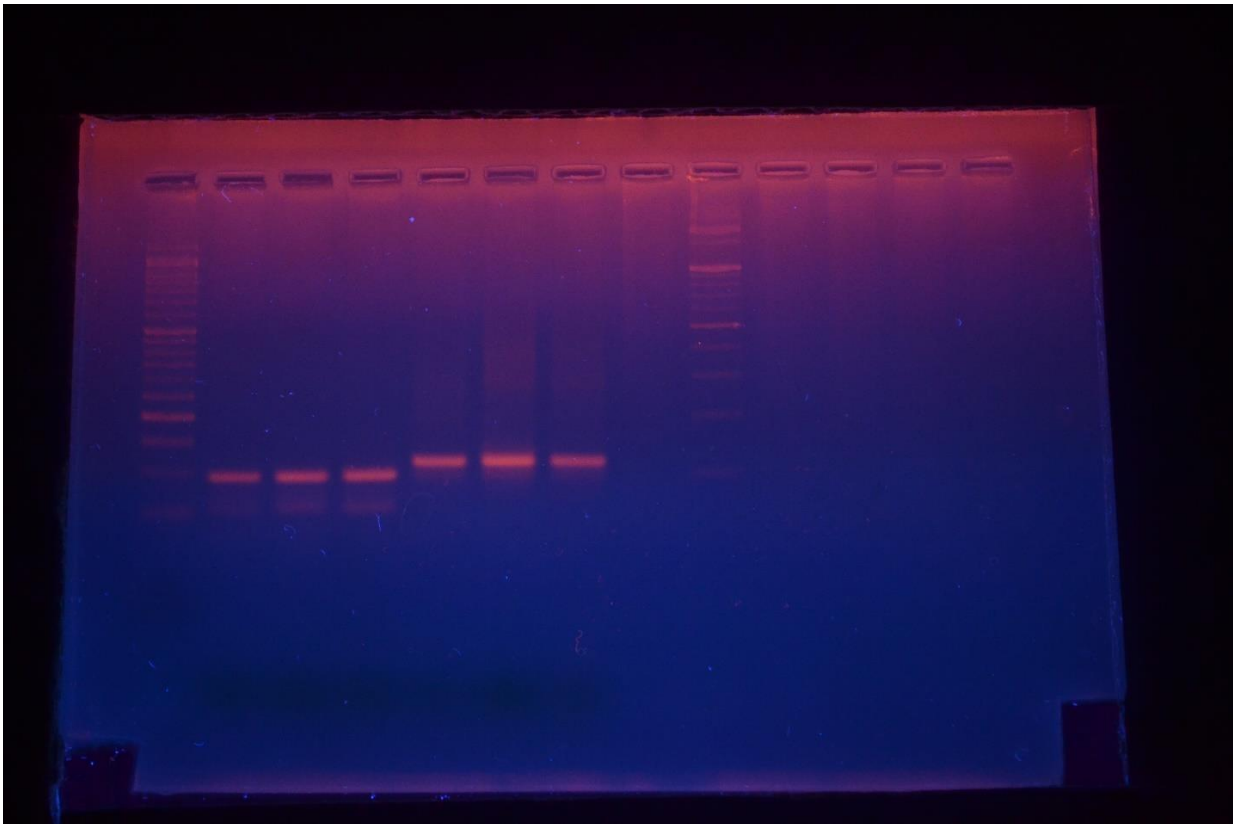

Supplementary Figure 3. Gel electrophoresis image showing 350 bp band indicating amplified region of domain III segment 6 of *vgsc* gene in *Ae. albopictus*. Lane M represent 100-1500 bp DNA ladder, Lane A, B, C, D, E represent amplified IIIS6 of *vgsc* gene and Lane F represent negative control.

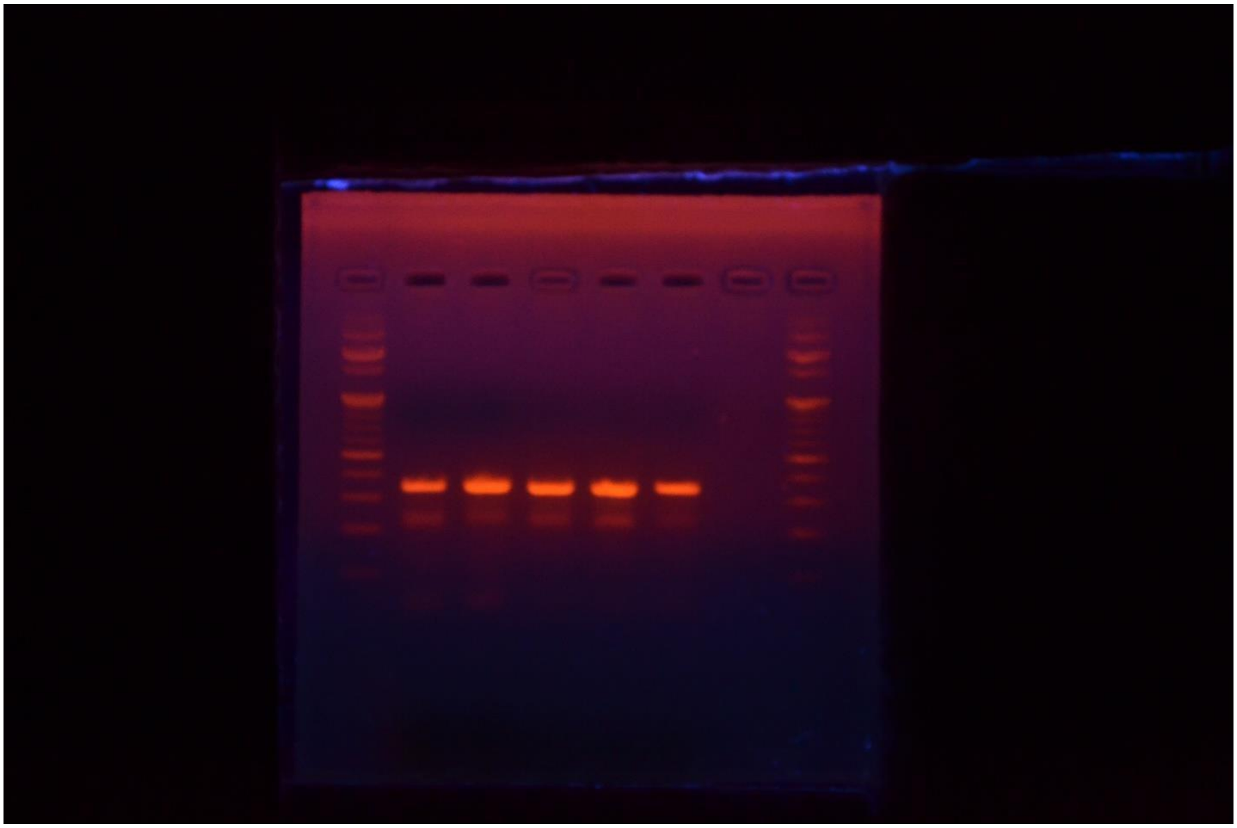

Supplementary Figure 4. Image of GenBank Accession no. confirmation.

MANAS PRATIM MODAK <rs_manas@nbu.ac.in>

GenBank accession OM421596-OM421597

gb-admin@ncbi.nlm.nih.gov <gb-admin@ncbi.nlm.nih.gov> Fri, Jan 28, 2022 at 5:23 AM
To: manas.p.modak@gmail.com, rs_manas@nbu.ac.in

Dear GenBank Submitter:

Thank you for your resubmission. Please find your assigned Accession Number(s) in the letter below.

We will attempt to add the correct annotation for you during the processing of your records. If we are unable to add the correct annotation, the following comment will be added to your record(s):

COMMENT GenBank staff is unable to verify sequence and/or annotation provided by the submitter.

Sequences flagged as unverified will not be included in NCBI BLAST databases.

For future submissions, please add the full and correct feature annotation when submitting. This will expedite the processing of your submission (including the assignment of accession numbers), and provide you with an additional tool to evaluate the quality of your nucleotide sequences. If you need assistance with adding annotation for your future submissions, contact our service desk at: info@ncbi.nlm.nih.gov.

Sincerely,

The GenBank Submissions Staff
Bethesda, Maryland USA
==

Dear GenBank Submitter:

Thank you for your submission of sequence data to GenBank, a contribution which will benefit the scientific community.

We have provided GenBank accession numbers for your nucleotide sequences:

BankIt2544073 1534Phe OM421596
BankIt2544085 1534Cys OM421597

The GenBank accession numbers should appear in any publication that reports or discusses these data, as it gives the community a unique label with which they may retrieve your data from our on-line servers. You may prepare and submit your manuscript before your accessions are released in GenBank.

Submissions are not automatically deposited into GenBank after being accessioned. Each sequence record is individually examined and processed by the GenBank annotation staff to ensure that it is free of errors or problems.

You have not requested a specific release date for your sequence data. Therefore, your record(s) will be released to the public database once they are processed. If this is not what you intended, please contact us as soon as possible with the correct release date.

Since the flatfile record is a display format only and is not an editable format of the data, do not make changes directly to a flatfile. For complete information about different methods to update a sequence record, see:

https://www.ncbi.nlm.nih.gov/Genbank/update.html
